# Newspaper Coverage of COVID-19 Vaccination Requirement for Organ Transplant

**DOI:** 10.1001/jamanetworkopen.2022.48800

**Published:** 2022-12-28

**Authors:** Marco Zenone, Timothy Caulfield

**Affiliations:** 1 Health Law Institute, Faculty of Law, University of Alberta, Edmonton, Alberta, Canada

## Abstract

This qualitative study summarizes newspaper reporting on views supporting or opposing policies requiring COVID-19 vaccination for organ transplant candidates in the US, the UK, and Canada.

## Introduction

Consensus exists among US transplant authorities to encourage COVID-19 vaccination in candidate organ recipients to minimize risk of posttransplant infection and increase odds of organ acceptance amid scarce organ resources.^1^ However, only 36% of US transplant centers require COVID-19 vaccination.^2^ While lifestyle and vaccination requirements are not new organ transplant conditions, the misinformation and public controversy surrounding COVID-19 policies may have led to misinformed or unbalanced portrayals of COVID-19 vaccines in the popular press.^3^ In this qualitative study, we examined newspaper coverage of the COVID-19 vaccination requirement for organ transplant to understand reporting portrayals.

## Methods

We searched newspapers in the US, Canada, and the UK, using the global news search engine Factiva (Dow Jones & Company) on May 23, 2022, for articles on the COVID-19 vaccination requirement for organ transplant. We confined our search to articles in English and keywords appearing in the headline or first paragraph to retrieve relevant results. Articles published on or before the search date were eligible for inclusion. We reviewed each article (n = 302) to ascertain whether it focused on COVID-19 vaccination as a transplant requirement, leaving 74 articles for inclusion in this analysis. We then analyzed the reasons against or in support of COVID-19 vaccination requirement for transplant. We reviewed, discussed, and agreed on the classification of these reasons. In accordance with the Tri-Council Policy Statement, ethical approval for this study was not required because the data collected were publicly available without privacy expectations. We followed the SRQR reporting guideline.

## Results

Across the 74 articles, 79.7% (n = 59) included an argument opposing and 94.6% (n = 70) included an argument supporting the vaccination requirement. Most arguments against it came from unvaccinated candidates awaiting an organ, their families, and organ donors. In some cases, opposition came from a political actor or institution. Arguments of support came from health care workers, center administrators, medical spokespersons, and ethicists. Such support often was in response to news of a prospective organ recipient who refused vaccination and was being denied an organ or a procedure.

Justifications for anti–COVID-19 vaccination requirement for organ transplant invoked personal values and beliefs, including freedom, rights, choice, and self-principles (55.9%); religious rights (40.7%); and opposition to vaccines that use or were developed at one time from aborted fetal cells or other human DNA (22.0%) (Table). Other reasons for refusal stemmed from misinformation or misconceptions surrounding COVID-19 vaccines or immunity, such as vaccine safety concerns (37.3%), belief that previous SARS-CoV-2 infection gave sufficient antibody protection (8.5%), and vaccine ineffectiveness (6.8%).

**Table.** Reported Arguments Opposing or Supporting the COVID-19 Vaccine Requirement for Organ Transplant (N = 74 articles)

Discourse type	Reason	No. of responses/No. of articles (%)	Example
Opposing requirements	Freedom, rights, choice, and self-principles	33/59 (55.9)	“It’s about standing up for our rights and understanding that we have a choice.”
	Religious rights	24/59 (40.7)	“Additionally, they oppose, under the religious laws established in the Book of Leviticus, vaccines that contain products from various animals it lists as ‘unclean.’”
	Vaccine safety concerns	22/59 (37.3)	“We’re being pressured to choose a shot that could kill him.”
	Vaccines using, or being developed at one time from, aborted fetal cells or human DNA	13/59 (22.0)	“I can’t support anything that has to do with abortion of babies, and the sanctity of life for me is precious.”
	Protection from previous COVID-19 infection	5/59 (8.5)	“A lot of scientists say I’m more safe from my natural antibodies. It’s more to do with the fact that they’re trying to force you to do something.”
	Vaccine ineffectiveness	4/59 (6.8)	“[Patient’s name] strongly believes that getting the vaccine should be a choice and doesn’t think it’s very effective in preventing infection.”
Supporting requirements	Posttransplant infection prevention, organ acceptance, and safety	70/70 (100.0)	“These are extremely high-risk patients….They are patients who, if they get COVID, are at profound risk of serious illness and death.”
	Consensus agreement on vaccine recommendation by scientific and medical groups	54/70 (77.1)	“Hospitals across the US have begun turning down vaccine refusals this year after the American Society of Transplantation and the International Society for Heart and Lung Transplantation recommended that all transplant patients should be vaccinated.”
	Vaccine and other lifestyle changes required for organ transplant eligibility before the COVID-19 pandemic	46/70 (65.7)	“Patients were already required to meet other stringent criteria to be considered for transplantation, including receiving inoculations against several illnesses, such as hepatitis B and influenza.”
	Organ scarcity and demand	40/70 (57.1)	“The organs are scarce, we are not going to distribute them to someone who has a poor chance of living when others who are vaccinated have a better chance post-surgery of surviving.”

Arguments supporting the requirement focused on the medical necessity of COVID-19 vaccination for successful organ transplant, explaining the importance of posttransplant infection prevention, organ acceptance, and safety for the organ recipient and others in recovery areas (100%) and the consensus agreement on vaccine recommendation by scientific and medical groups (77.1%) (Table). These arguments also contextualized vaccinations and other lifestyle factors as conditions of organ transplant eligibility even before the COVID-19 pandemic (65.7%). Scarcity of organs, number of prospective recipients, and need to maximize chances of successful transplants through protective factors, such as vaccination requirement, were also elucidated (57.1%).

## Discussion

Results demonstrated a false balance, referring to newspaper reporting that gave an “equal platform… to opposing views despite one view holding expert and evidentiary support” regarding COVID-19 vaccination requirement for organ transplant.^4^^p(258)^ A study limitation was using country-specific newspapers only, not grey literature or non-text-based sources.

Arguments for opposing vaccination were rooted in personal beliefs or misinformation about the systemic, medical, and ethical reasons^5^ for the requirement. While views of health care workers and other medical actors countered this frame, false balance may distort public perception on noncontroversial subjects when correct information is concurrently presented.^6^ Thus, reporting on COVID-19 vaccination requirement for organ transplant, which elevated the perspectives of vaccine refusal based on personal belief and misinformation, may contribute to misperceptions about a consensus medical viewpoint and create controversy where none exists.

## Notes

Supplement.
Data Sharing Statement
