# Supplementary material for: Newspaper Coverage of COVID-19 Vaccination Requirement for Organ Transplant
Source: JAMA Netw Open. 2022 Dec 28;5(12):e2248800. doi: 10.1001/jamanetworkopen.2022.48800 (PMC9857094; doi:10.1001/jamanetworkopen.2022.48800)
Supplement: Supplement. — Data Sharing Statement [file jamanetwopen-e2248800-s001.pdf]

Data Sharing Statement

Zenone. Newspaper Coverage of COVID-19 Vaccination Requirement for Organ Transplant. *JAMA Netw Open*. Published December 28, 2022. doi:10.1001/jamanetworkopen.2022.48800

Data
Data available: Yes
Data types: Other (please specify)
Additional Information: The data is available upon reasonable request from the corresponding author.
How to access data: Zenone@ualberta.ca
When available: With publication

Supporting Documents
Document types: None

Additional Information
Who can access the data: Anyone requesting the data.
Types of analyses: Any purpose.
Mechanisms of data availability: By email request.
